# Biodegradable Chitosan-Based Membranes for Highly Effective Separation of Emulsified Oil/Water

**DOI:** 10.1089/ees.2022.0254

**Published:** 2022-12-13

**Authors:** Peng Wan, Xuanning Yang, Qinhua Feng, Shuyu Shi, Baolin Deng, Lina Zhang

**Affiliations:** ^1^ Department of Biomedical, Biological and Chemical Engineering, University of Missouri, Columbia, Missouri, USA.; ^2^ Guangdong Provincial Engineering and Technology Research Center for Water Affairs, Big Data and Water Ecology, Shenzhen Water Planning & Design Institute Co., Ltd., Shenzhen, China.; ^3^ School of Environmental Science & Engineering, Southern University of Science and Technology, Shenzhen, China.; ^4^ College of Chemistry and Molecular Sciences, Wuhan University, Wuhan, China.; ^5^ Department of Civil and Environmental Engineering, University of Missouri, Columbia, Missouri, USA.

**Keywords:** biodegradable material, chitosan, dopamine, membrane separation, oil/water emulsion

## Abstract

Efficient separation of oil droplets from oil/water emulsions is necessary for many energy and food industrial processes and for industrial wastewater treatment. Membrane microfiltration has been explored to address this issue because it is simple to operate and low in cost. However, filtration of oil droplets with a size around or less than 1 μm is still a major challenge. Furthermore, the fabrication process for polymeric membranes often uses hazardous organic solvents and petroleum-derived and nonbiodegradable raw materials, which pose additional environmental health and safety risk. In this study, we examined the use of chitosan-based membranes to efficiently remove oil droplets with an average diameter of ∼1 μm. The membranes were fabricated based on the rapid dissolution of chitosan in an alkaline/urea solvent system at a low temperature, thus avoiding the use of any toxic organic solvent. The chitosan membranes were further modified by dopamine and tannic acid (TA). The as-prepared membrane was characterized in terms of surface morphology, pore size distribution, and mechanical strength. The membrane performance was evaluated on a custom-designed crossflow filtration system. The results showed that the modified chitosan membrane with dopamine and TA had a water flux of 230.9 LMH at 1bar transmembrane pressure and oil droplet rejection of 99%. This water flux represented an increase of more than 10 times when compared with the original chitosan membrane without modification. The study also demonstrated excellent antifouling properties of the modified membrane that could achieve near 100% water flux recovery.

## Introduction

Water shortage has become a critical issue in many regions of the world due to rapid population and economic growth (Shannon et al, 2008; Xiong et al, 2020; Zhu et al, 2020a, 2020b). The water supply sustainability and environmental quality are closely coupled with energy production from fossil fuels and water pollution control. A large quantity of oily water was generated from various industries and daily human activities. To maintain water and energy sustainability, therefore, it is critical to have efficient technologies of purifying oily water in various industrial and domestic wastewater streams.

Often, the phase-separated oil/water waste streams could be treated via conventional strategies such as by API oil separators, weir skimming, air floatation, and gravity separation (Liu et al, 2019). Emulsified oil/water waste streams are more difficult to handle; their treatments are mostly based on de-emulsification, coagulation, and absorption. These oil emulsion separation approaches, however, suffered from low separation efficiency and generation of secondary pollutions (Major et al, 2012).

Membrane technology has been increasingly explored to separate oil/water emulsions because of its high separation performance and easy operation (Islam et al, 2017; Wang et al, 2015). However, it remains quite a challenge to separate emulsions with the size of oil droplets approximately at or less than 1 μm, which required the use of membranes with nano-sized pores and high mechanical strength (Panatdasirisuk et al, 2017). Particles at around 1 μm in diameter are at their minimal for particle–particle interactions and thus most difficult to coagulate to form larger sizes (Yao et al, 1971).

Membranes for oil/water separation could be ceramic or polymeric (Ahmed et al, 2007; Jamshidi Gohari et al, 2015; Wang et al, 2014; Zhu et al, 2014). Ceramic membrane is durable, but has low separation efficiency, high cost, and complex membrane production process. Hydrophobic polymeric membranes have intrinsically low water permeability, so they often need to be modified to enhance their hydrophilicity by blending, coating, or layer by layer modification and grafting. To have a membrane process that is sustainable and green to the environment, it is desirable to develop a membrane that begins with sustainable materials, is manufactured by green processes, and is biodegradable upon its usage and disposal.

Chitosan, an alkaline polysaccharide derived from chitin residing in shrimp and other crustacean shells (Smets and Rüdelsheim, 2018), appears to be a proper choice to make a biodegradable membrane. Every year, about 8 million tons of seafood waste are produced from shrimp, crab, and lobster shells, some of which are disposed of in landfills or to the sea (Yan and Chen, 2015). Chitin and chitosan could be utilized in different areas due to their innate biodegradability, biocompatibility, and nontoxicity (Fang et al, 2017; Xiong et al, 2020). They differ in their degrees of acetylation; the deacetylation of the chitin results in formation of chitosan.

It has been well established that chitosan can be dissolved in a dilute acid, and introducing an acidic chitosan solution to an alkaline coagulation bath regenerates chitosan hydrogels (Duan et al, 2015; Gang et al, 2010). This process of hydrogel formation has been widely used in areas such as food science, medicine, and biomaterials (Cao et al, 2018). One shortcoming with the chitosan hydrogel based on acid dissolution is its relative weak mechanical properties (Wen et al, 2017). To address this issue, a number of studies explored chitosan assembly with chemical crosslinking, nanofillers reinforcement, and solid supports for the purpose of concentrating glycopeptides (Fang et al, 2014; Li et al, 2012; Wen et al, 2017; Xiong et al, 2013). Alternatively, a number of studies used chitosan to modify other materials so better oil/water separation efficiencies could be achieved (Doan et al, 2021; Krishnamoorthi et al, 2022).

Currently, chitosan films/membranes are mostly developed from regenerated hydrogel in acidic solution, geared to biomedical applications (Azad et al, 2004; Jin et al, 2011). Different from the conventional acid chitosan dissolution, a new platform technology based on alkali/urea aqueous solutions for direct dissolution of cellulose-like structure at low temperature was developed at Wuhan University (Duan et al, 2015; Isobe et al, 2013; Liu et al, 2013; Qi et al, 2009). Membranes developed from the alkaline/urea dissolution are stronger and could potentially be tuned to have a specific service life (Shi et al, 2020). The addition of urea resulted in an enhanced dissolution rate and solubility during the freezing-thaw process because of the formation of urea inclusion complex.

Cellulose, chitin, and chitosan hydrogels have all been constructed via alkali/urea aqueous system for making biomaterials and adsorbents (Chang et al, 2011; Duan et al, 2015; Gong et al, 2016; Liang et al, 2017; Shi et al, 2015). It is largely unknown, however, if a chitosan film formed from an alkaline solution can be used as a separation membrane for water treatment. It is thus inspirational to explore the possibility of applying a chitosan film developed by the alkaline solvent platform as a separation membrane suitable for oily water treatment. In addition, while there are reactive functional groups on chitosan molecule that can easily form hydrogen bonds, chitosan film can be hydrophobic, probably resulted from the spatial orientation of chitosan fibers (Hubbe, 2019). Surface modification by hydrophilic moieties should enhance the membrane hydrophilicity and filtration performance because of changes of the somewhat hydrophobic nature in chitosan (Koyano et al, 2000).

Approaches to increase surface hydrophilicity are mostly based on introducing hydrophilic groups. Derived from adhesive components in mussels, dopamine was of the chemicals widely used for surface modification, it could self-polymerize to form thin films onto various inorganic and organic materials (Lee et al, 2007). Catechol compounds in dopamine structure served as binding agents (Wan et al, 2019). The polymerization process itself, however, would decrease the population of reactive amine and catechol groups in dopamine. Tannic acid (TA) is another chemical, found in plant-extracts, which possesses strong binding with dopamine through covalent and noncovalent interactions (Shih et al, 2018). It is a low-cost polyphenol readily obtainable from tea leaves, oak wood, nettle, and Chinese galls (Dhand et al, 2016). The abundant catechol or galloyl groups could result in strongly hydrophilic surfaces (Zhang et al, 2016). Our preliminary tests showed that the dopamine could facilitate coatings of TA onto chitosan membranes.

The goal of this research is to fabricate flexible chitosan membranes by exploiting the rapid and high dissolution of chitosan fibers using alkali/urea aqueous solution as a solvent, and to demonstrate membrane's potential application for oily wastewater purification. Dopamine was applied to facilitate the modification of membranes with TA because of its known tendency of self-polymerization and interactions with TA. The membrane performance was characterized in terms of its water flux improvement and antifouling property. The particle size of oil-in-water emulsion in the feed was centered around 1 μm, a size range most difficult to remove by other approaches.

In contrast to the use of hazardous solvents for fabrication of traditional polymeric membranes, such as N-methyl-2-pyrrolidone, dimethylacetamide, and dimethylformamide (Kim and Deng, 2011; Tian and Jiang, 2008; Wan et al, 2020), this biodegradable membrane could be fabricated after low-temperature alkaline dissolution/dispersion of chitosan, thus the material and the process both are considered green and superior to the fabrication of traditional polymeric membranes. To our knowledge, this is the first study on the separation of oil/water emulsions by the chitosan membrane, fabricated via a green alkali/urea aqueous solvent system and modified by the bio-inspired sustainable materials, dopamine, and TA.

## Materials and Methods

### Materials

Chitosan (200–400 mPa.s), Span 85, Tween 80, and TA were purchased from Aladdin Reagent Co., Ltd. (Shanghai, China). Tris-(hydroxymethyl)aminomethane (99.8+%) (Tris) and dopamine hydrochloride were obtained from Sigma-Aldrich. The oil/water emulsion with oil droplet size around 1 μm was prepared by a reported method, in which hydraulic oil (8.0 g) was mixed with 1.0 g Tween 80 and 1.0 g Span 85 for 30 min, followed by its dispersion into 2,500 mL DI water (Chang et al, 2014).

### Dissolution of chitosan in aqueous alkaline/urea solvent

The aqueous solution containing LiOH/KOH/urea/H_2_O in the ratio of 4.5:7:8:80.5 by weight was used as a solvent for chitosan dissolution under stirring. The formed suspension was transferred to and stored in the refrigerator at −30°C until frozen. Then, the frozen sample was fully thawed and stirred extensively at room temperature. The as-prepared solution was centrifuged at 10,000 rpm for 5 min at 5°C to remove bubbles and impurities. A series of transparent solutions were obtained with a chitosan concentration of 2%, 3%, 4% 5%, or 6%.

### Preparation of chitosan membranes

**FIG. 1.**
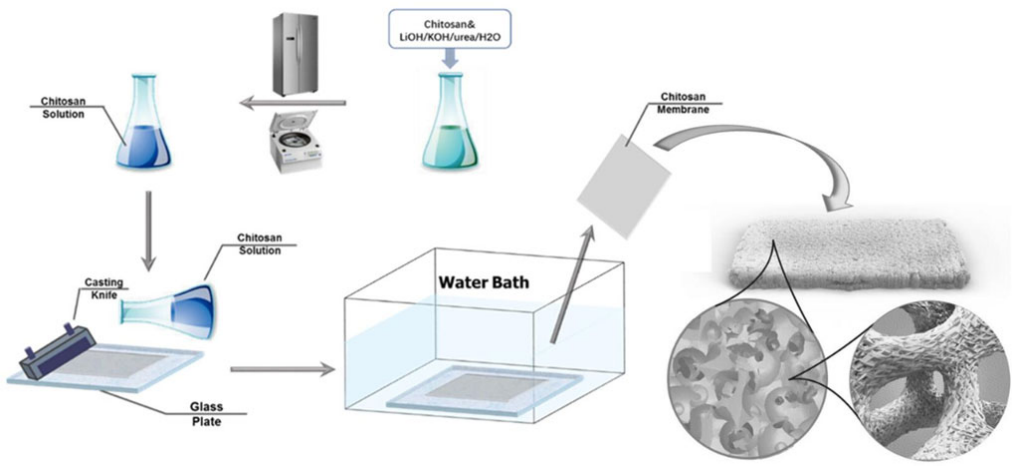
An illustration of chitosan membrane fabrication process.

The transparent chitosan solution in the alkaline/urea solvent was spread on a glass plate, which was casted to be at ∼100 μm in thickness by a casting knife. Then the glass plate was immediately transferred to hot water at 40°C and soaked for 30 min, during which the solution was transformed to form chitosan membrane. After the removal of residual solvent by washing with ethanol and DI water thoroughly, the membrane was stored in DI water for further testing. A schematic representation of the chitosan membrane fabrication process is shown in Fig. 1.

### Modification of chitosan membranes

**FIG. 2.**
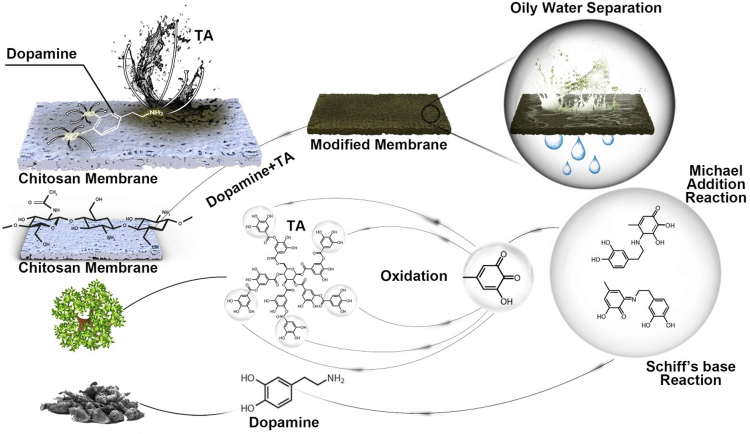
An illustration of chitosan membrane modification process with dopamine and tannic acid (TA).

First, the chitosan membrane was treated by a 0.2 wt % dopamine/buffer solution (Tris-HCl, pH 8.5) for 1 h so that a dopamine coating could form on the membrane surface due to its self-polymerization. This membrane, named 1PDA, was then washed by ethanol and DI water three times. The membrane was soaked in DI water for at least 30 min afterward for the exchange of ethanol out of the membrane. The modified membrane was then immersed in a 0.2 wt % TA buffer solution (Tris-HCl, pH 8.5) for 3 h/6 h/9 h/12 h/18 h/24 h. These membranes were named 1PDA 3TA, 1PDA 6TA, 1PDA 9TA, 1PDA 12TA, 1PDA 18TA, and 1PDA 24TA, respectively. The chitosan membrane modification mechanism is illustrated in Fig. 2.

### Membrane characterizations

The chitosan membranes were characterized by a high-resolution scanning electron microscope (ZEISS, MERLIN, Germany) for their morphology and porous structure. The software ImageJ was used to analyze the SEM images for the estimate of the average membrane pore size. A 3H-2000 PB membrane pore size analyzer (BeishiDe Instrument Ltd.) was applied for the pore size distribution analysis. Dynamic light scattering (DLS) was used to determine the particle size of oil droplets in water-in-water emulsions (ZetasizerNano ZS90; Malvern Instrument Ltd.). Characterization of the membrane hydrophilicity was conducted by using a video contact angle system (VCA-2500 XE, AST products, Billerica, MA), based on the sessile drop method (Yin et al, 2016; Yin et al, 2012). The tensile strength tests for chitosan membranes were conducted on a universal tensile testing machine (CMT 6104).

The membrane water flux and oil emulsion rejection were evaluated on a crossflow filtration system. The membrane was compacted by DI water at 15 psi for 2 h to stabilize the system before each test. The permeating flux was determined by using a LabVIEW automated system (National Instruments) coupled with the Ohaus digital balance to weigh and record the amount of permeate solution as a function of time. The concentration of oil emulsions was determined by UV-visible spectrophotometry at 376 nm (Arshadi et al, 2019; Chang et al, 2014), which recorded absorbance values or the relative light transmission intensity. Accurate determination of turbidity or oil concentration in the colloidal system by this approach needs to account for changing particle size distribution in the feed and filtrate, proper light sources, and aggregation kinetics (Barbaro et al, 1991; Liu et al, 2007).

However, turbidity measurements appeared to be quite consistent with particle size analysis for oil-in-water emulsions (Reddy and Fogler, 1981), thus the approach is adequate for assessing relative performance of membranes. The flux and rejection were calculated by Equations (1) and (2), respectively:

(1) $$J=\frac{V_{p}}{A\cdot t}$$

(2) $$R=\left(1-\frac{C_{p}}{C_{f}}\right)\times 100$$

where J (L/m^2^h) represents the flux of permeate solution, V_p_ (L) the permeate volume, A (m^2^) the membrane surface area, t (h) the time, R the rejection ratio, C_p_ the oil concentration in the permeate, and C_f_ the oil concentration in the feed solution. The flux recovery ratio F_RR_ is defined and calculated by Equation (3), in which (*J_w, i_*) and (*J_w, i-1_*) are the pure water flux after and before the i^th^ filtration of the oil emulsion following the pure water filtration.

(3) $$F_{RR,i}\left(\%\right)=\frac{J_{w,i}}{J_{w,i-1}}\times 100$$

## Results and Discussion

**FIG. 3.**
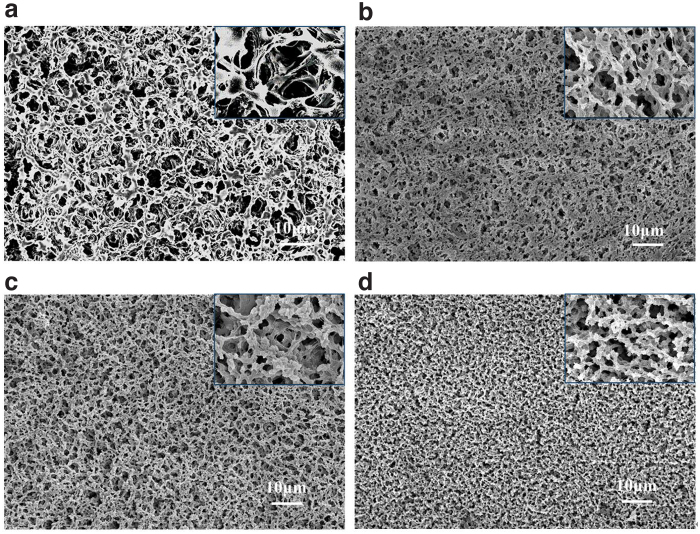
The morphologies of chitosan membranes fabricated at **(a)** 3%, **(b)** 4%, **(c)** 5%, and **(d)** 6% chitosan dope solution. (magnification of 1 kx and magnification of 10 kx).

As shown by Fig. 3 and Supplementary Figure S1, all chitosan membranes prepared under different chitosan concentrations exhibited a homogeneous microporous structure desirable for separation membranes. The pore size of the chitosan membrane decreased with an increasing chitosan concentration. This observation is consistent with the general understanding of the system: by immersing the alkaline chitosan dope solution in water, the alkaline-urea complex shell on the chitosan was destroyed, resulting in self-aggregation of the chitosan chains in parallel to form tangled nanofibers (Zhu et al, 2017). The destruction of the alkaline-urea shell that encases the chitosan chain is likely facilitated by both the temperature increase following the immersion and the decrease of alkaline-urea concentration upon the solvent exchange.

Once the chitosan chains are destabilized, they begin to aggregate to form fiber bundles, which is a dynamic process controlled by the original chitosan concentration, the rate of heat exchange and temperature homogenization, and dispersion of alkaline-urea ions out of chitosan dope and water into the chitosan gel matrix. Subsequently, a network of fiber bundles is generated with the aggregated chitosan fibers serving as a pore wall and openings left from the process as pores. The pore size decreased as the original chitosan concentration was increased from 3% to 6%. It is worth noting that a higher chitosan concentration did not result in fiber bundles with larger diameters but a denser fiber network. The observation suggests that the destabilized chitosan chains might not have high mobility because of their tangled structure so the aggregation occurred somewhat locally for fiber bundle formation but across the whole casted membranes.

**FIG. 4.**
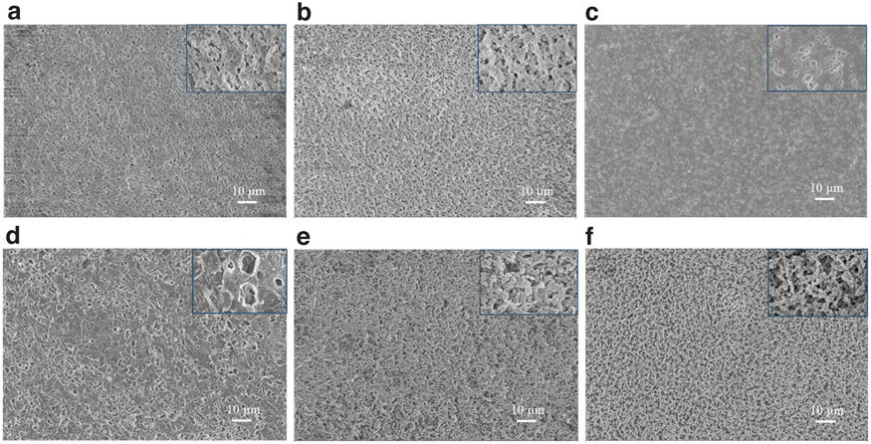
The morphologies of modified chitosan membranes: **(a)** 1PDA 3TA, **(b)** 1PDA 6TA, **(c)** 1PDA 9TA, **(d)** 1PDA 12TA, **(e)** 1PDA 18TA, **(f)** 1PDA 24TA. (magnification of 2 kx and magnification of 20 kx).

For the fabricated chitosan membranes discussed above, the water flux was higher when a dope solution of lower chitosan concentration was used. While a higher water flux is more desirable for application, unfortunately, the mechanical strength of the membrane was decreased with lowering chitosan concentration. To illustrate, our preliminary experiments showed that the mechanical strength of the membrane prepared for 3% chitosan or lower was very low and could be broken during testing. Therefore, the membrane prepared using 4% chitosan was selected for studying the impact of chitosan membrane modification. By treating with 0.2% dopamine for 1 h, the dopamine-modified membranes showed comparable morphologies to the original membrane (Fig. 4 and Supplementary Fig. S2), suggesting that the dopamine coating on the membrane surface was thin and homogeneous.

The *in situ* adhesion and polymerization of PDA onto the membrane has been attributed to the formation of hydrogen bonding, Michael-type addition from o-quinones, and π–π bonding (Wan et al, 2019). With further treatment by 0.2% TA for a period ranging from 3 h to 24 h, denser and more rugged topographical morphologies emerged as the dip-coating time was increased. TA extracted from plants has a poorly defined structure but abundant catechol or galloyl reactive moieties. Its relatively large molecule weight (MW = 1,701 for pure TA) allows it to cover the membrane surface (Xu et al, 2018) and crosslink with amine and other functional groups on chitosan and PDA.

It is known that amino groups on dopamine and chitosan could react with the oxidation products of TA through Michael addition and Schiff base reaction (Wang et al, 2018; Yang et al, 2019). TA had a significant impact on the formation of the distinct hierarchical structures and brought many hydrophilic functional groups onto the dopamine-modified membrane surface.

The mechanical strength of the chitosan membrane was found closely linked to the chitosan concentration in the dope used for the membrane fabrication (Supplementary Fig. S3 in Supporting Information). The ultimate tensile stresses of the chitosan membranes were 0.167, 0.20, 0.52, and 0.83 Mpa for the membranes prepared from 3%, 4%, 5%, and 6% of chitosan, respectively. The relatively high stress-strain characteristics prepared from the alkaline/urea solvent system might be attributed to the regenerated nanofibers that had a compact and homogeneous architecture and strong networks (Duan et al, 2015). The tensile stress of the chitosan membrane increased with increasing chitosan concentrations. The membranes prepared from a higher chitosan concentration are more compact with strong fiber networks and smaller pores as shown in Fig. 3. It is known that the pore size reduction could improve mechanical properties of the composite hydrogel (Huang et al, 2017).

Once the chitosan membrane with a proper structure was developed, we initiated a new modification process by treating the membrane with both dopamine and TA to increase its hydrophilicity, aiming to demonstrate the potential application of chitosan membrane for oily water treatment. The membrane surface hydrophilicity was assessed by contact angle measurements, with the results presented in Supplementary Fig. S4 (Supporting Information). The 4% chitosan membrane originally had a water contact angle of 45.9°, and upon its modification by dopamine, the contact angle was decreased to 28.7°. Further treatment of the membrane with TA for different durations resulted in membranes all exhibiting a water contact angle of 0°. While there are hydroxyl and amine functional groups on chitosan, it is well known that the chitosan surface is somewhat hydrophobic (Koyano et al, 2000).

Reaction and coating with dopamine increased its surface hydrophilicity as indicated by the contact angle decrease of around 17°, but the TA coating had the most dramatic effect, transforming the chitosan membranes to a highly hydrophilic surface due to the presence of abundant hydrophilic hydroxyl groups in the TA. This is not surprising because even a small amount of TA coating could switch the hydrophobic pristine membrane to superhydrophilic ones (Xu et al, 2018). In another study, a poly (vinylidene fluoride) membrane was treated by dopamine and TA, resulting in a contact angle of 0°, on the modified membrane surface (Yang et al, 2019), which is consistent with what we observed with the chitosan membrane.

**FIG. 5.**
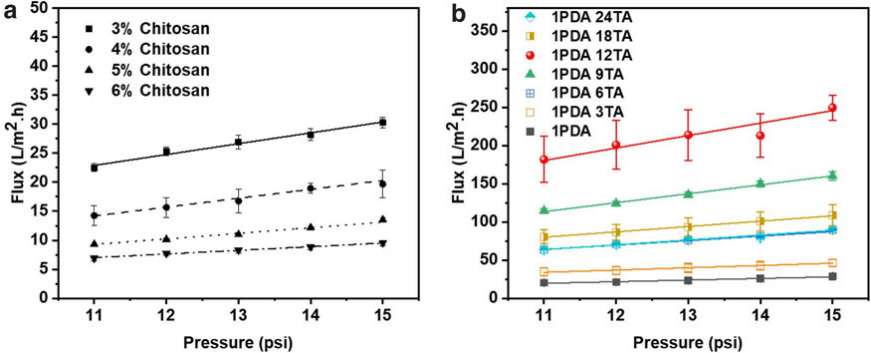
**(a, b)** Pressure-dependent pure water fluxes of chitosan membranes.

The pure water fluxes of chitosan membranes under different pressure are presented in Fig. 5a. As expected, the water flux increased with increasing operation pressure for all membranes. At 15 psi cross-membrane pressure, the pure water fluxes are 42.9, 28.6, 13.6, and 9.5 L/m^2^.h for membranes prepared with 3%, 4%, 5%, and 6% of chitosan, respectively. The pure water flux of the membrane depended on membrane porosity, pore interconnection, pore size, and size distribution, as well as the surface chemistries (Wan et al, 2017b). The chitosan membrane structure was decided by the nature and concentration of dope solution and formation process. As the chitosan concentration increases, the aggregation of chitosan fiber occurs more readily by the solvent exchange and inclusion complex disruption, leading to formation of chitosan membranes with denser structure. The result is consistent with the observation that the higher the dope solution the lower the pure water flux.

When the chitosan membrane, prepared with 4% chitosan concentration, was modified with dopamine and TA, the pure water flux was dramatically increased (Fig. 5b). The water flux of the membrane modified by PDA alone is 25.9 LMH at 1bar, which represents a 34% increase when compared with 19.3 LMH observed for the original chitosan membrane. The water fluxes were increased to 45.1, 86.6, 157.4, 230.9, 106.2, and 86.9 LMH at 1 bar for 1PDA 3TA, 1PDA 6TA, 1PDA 9TA, 1PDA 12TA, 1PDA 18TA, and 1PDA 24TA, respectively.

A similar trend was observed with other membranes of different chitosan concentrations, when being modified with PDA and TA. It is interesting to note that a clear optimal time for TA modification could be determined (Fig. 5b): the water flux increased with increasing treatment time by TA from zero to 12 h, but further increases in the coating time resulted in a decrease in water flux. The water flux of 1PDA 12TA (the membrane with 12 h treatment by TA) was the highest, representing a more than 10 times of the flux in comparison with the original chitosan membrane.

The pure water permeance of the 4% chitosan membrane was low at ∼20 L/m^2^-h-bar, which was consistent with the low surface hydrophilicity (see Fig. S4). The dopamine-modified membrane had an increased water flux due to the increase in hydrophilicity as indicated by a decreased contact angle. The increase is much more effectively imparted by the TA coating because of the galloyl moieties, as suggested by the over 10 times water flux increase for the membrane coated for 12 h. Excessive coating beyond 12 h, however, has likely resulted in too much accumulation of TA, thus leading to pore blocking and decrease in pure water fluxes. There was therefore a need to balance the improvement of hydrophilicity and reduction of pore size during the TA coating process (Xu et al, 2018).

**FIG. 6.**
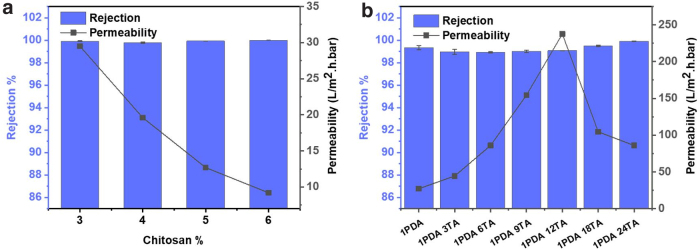
**(a, b)** The rejections and permeabilities of chitosan membrane samples.

**FIG. 7.**
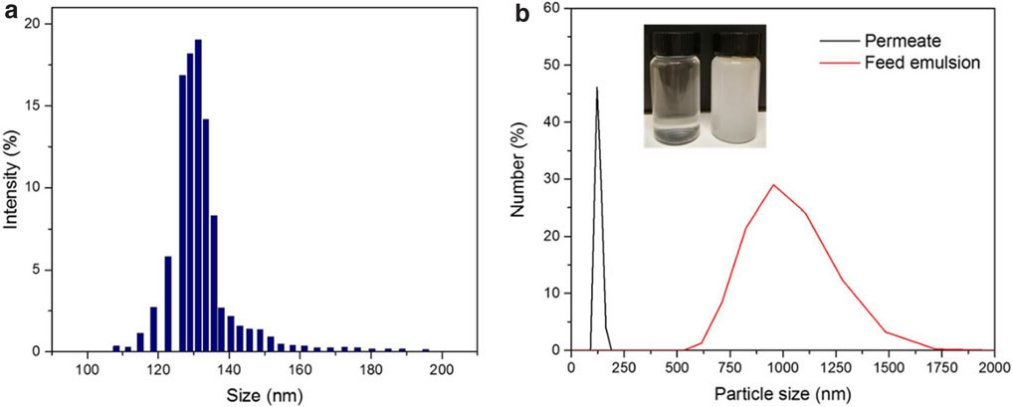
**(a)** Pore size distribution of 4% chitosan membrane; **(b)** The measured particle size distribution of surfactant stabilized oil-in-water emulsion of permeate solution and feed emulsion.

The oil-in-water emulsion rejections were tested for each of the fabricated chitosan membranes, including the unmodified and modified ones. The oil rejections by chitosan membranes prepared with different concentrations (Fig. 6a) and by those modified with dopamine and TA (Fig. 6b) were all higher than 99%. The observation could be explained by the pore size of the membranes and the particle size of the oil-in-water emulsions. As indicated by the pore size distribution of 4% chitosan membrane shown in Fig. 7a, the pore diameter is between 100 and 200 nm. The particle size of oil-in-water emulsion in the feed was between 500 and 1,750 nm, centered around 1,000 nm (Fig. 7b). The diameter of oil-in-water emulsion was significantly bigger than the membrane pore size; as a result, an efficient rejection of the oil-in-water emulsions with a size around 1 μm could occur.

The DLS measurements showed the presence of some particles smaller than 200 nm in the permeate, suggesting that when the oil-in-water emulsion particles be smaller than the membrane pore size, their leakage to the permeate is still possible. However, that fraction is tiny, at less than 1% of the oil in the feed water, although the number concentration is high.

The primary mechanisms for the oil droplet rejection may include size exclusion and repelling by the super oleophobic surface. The effect of electrostatic interactions may be insignificant because the membrane pore diameter (in the range of 100–200 nm) is much larger than the normal electrical double layer thickness. The molecular size of TA is in a range of nanometers, so during the TA coating, TA could infiltrate into the porous structure and thus result in the formation of hydrophilic coating on the membrane surface as well as in the pores. The TA coating at a monolayer would not significantly decrease the pore size. However, as indicated by the Fig. 6b, membranes treated beyond the 12 h of optimal time had a decreased water flux, which may be caused by partial pore blocking.

Overall, it appeared that the size exclusion would be the dominant mechanism for the oil droplet rejection because all membranes, regardless of surface modification by dopamine alone or with further TA treatment, had high (>99%) but comparable rejection. Nevertheless, the contribution of the super oleophobic surface imparted by the TA layer could still play a role in the maintenance of the high rejection considering the much higher water flux with the modified membrane with high hydrophilicity. Further work is needed to quantify this contribution.

It should be noted that while the surface modification implemented in this study could increase water flux for more than 10 times when used for oily wastewater treatment when compared with the original chitosan membrane, even the modified chitosan membrane does not have a water permeability as high as some commercial polyvinylidene difluoride and polysulfone membranes (Ismail et al, 2020; Yi et al, 2019). Nevertheless, fouling for the hydrophobic polymeric membranes is often a major problem (Yi et al, 2019). Also the traditional polymeric membranes or ceramic membranes used for oil-in-water emulsions are often expensive for production, might involve the use of hazardous solvents for fabrication, and are difficult for disposal. Chitosan-based membranes as developed here from the alkaline-urea platform have much less of these concerns when used for oily wastewater treatment.

Chitosan can be dissolved in an acidic solution as well, and solubilized chitosan has been explored to make a membrane via a multiple coating process for the removal of dye molecules (Long et al, 2020). However, the weak mechanical strength is a disadvantage as discussed earlier (Duan et al, 2015). One study explored coating chitosan solubilized in acid onto polyphenylene sulfide membrane for oil separation (Huang et al, 2019), where the mechanical strength of the chitosan gel is not a concern.

Fouling prevention and control are necessary to apply membrane separation for oil emulsion treatment. For the membranes modified with dopamine and TA in this study, its antifouling performance was evaluated by using the cyclic filtration tests. In brief, after compaction, the membrane was exposed to DI water for 20 min to obtain the pure water flux (J_w0_), then the feed solution was switched to the oil-in-water emulsion for 30 min. After the membrane was cleaned with a pure water flush for 10 min, the feed was changed to DI water again for another 20 min, completing one cycle. Initial fluxes in all the fouling tests were set approximately the same.

**FIG. 8.**
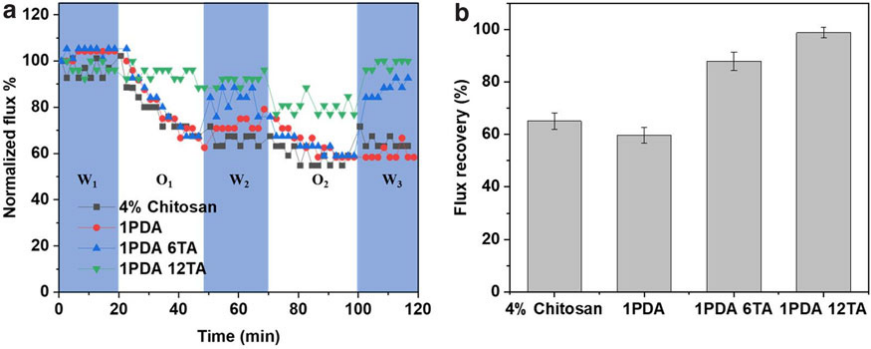
**(a)** Time-dependent fluxes through the membranes during a cyclic filtration process at 25°C (W: filtration by pure water; O: filtration by oil-in-water emulsions); **(b)** Water flux recovery for membrane samples.

The results (Fig. 8a) indicated that the relative flux of 1PDA was similar to that of 4% chitosan membrane, while the relative fluxes of 1PDA 6TA and 1PDA 12TA were significantly higher. The relative pure water flux could reach 100% after 2 cyclic filtrations for 1PDA 12TA, which was higher than that of 1PDA 6TA. This could be contributed to the superhydrophilic characteristics of TA and PDA coated onto the membrane surface. A higher amount of hydrophilic polymer on the membrane could endow a better antifouling property to oil, which was consistent with reported results when different amounts of monomers were applied on membranes to tailor the antifouling property (Wan et al, 2017a).

Water flux recoveries were summarized for the tested membranes as shown in Fig. 8b. The modification of dopamine alone could not improve the water flux recovery, while the flux recovery ratio was increased by coating of TA and PDA. The 1PDA 12TA showed the best flux recovery ratios among the tested membranes.

## Conclusions

Robust chitosan-based membranes were successfully fabricated in an alkali/urea aqueous solvent system, followed by dopamine and TA modifications. The chitosan membrane became denser as the concentration of chitosan in the casting solution was increased. For oil water emulsions, the chitosan membranes exhibited excellent separation performance with higher than 99% oil droplet rejection. The flux of the chitosan membrane was enhanced by more than 10 times following the modification with dopamine and TA when compared with the original chitosan membrane. The modified chitosan membrane could also achieve nearly 100% membrane flux recovery and possessed excellent antifouling properties. This work offered a simple, low cost, and environment friendly method for the fabrication and modification of a chitosan membrane capable of effective oil/water separation.

In particular, only biodegradable chemicals were used for the membrane fabrication/modification, and the chitosan membrane itself is biodegradable upon disposal, so the approach has merits from the life cycle assessment perspectives. Under the context of increasing concerns with the microplastics in the ocean and terrestrial aquatic system, using green chemistry for a biodegradable functional membrane helps maintain sustainability, by avoiding the use of persistent, petroleum-based materials for membrane fabrication.

## Supplementary Material

Supplemental data

Supplemental data

Supplemental data

Supplemental data
