# Supplementary figures and images for: Biodegradable Chitosan-Based Membranes for Highly Effective Separation of Emulsified Oil/Water
Source: Environ Eng Sci. 2022 Dec 13;39(12):907–17. doi: 10.1089/ees.2022.0254 (PMC9807252; doi:10.1089/ees.2022.0254)

**
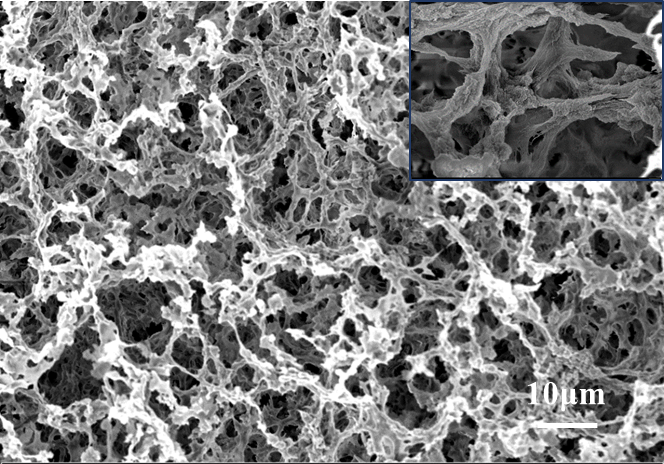
**

**Figure S1.** The morphology of 2% chitosan membrane (magnification of 1 kx and magnification of 10 kx).

Supplement: Supplemental data [file Suppl_FigS1.docx]

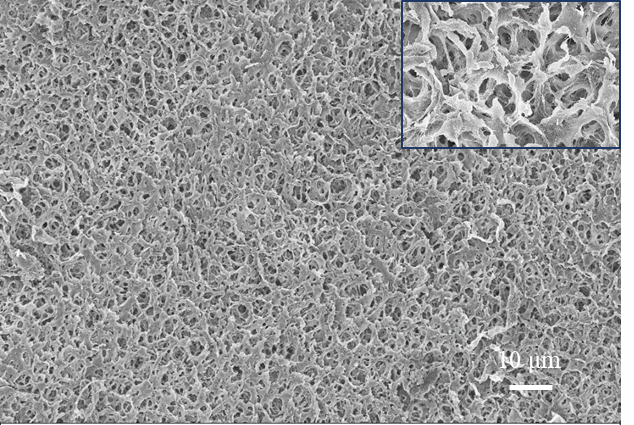


**Figure S2.** The morphology of 1PDA membrane sample (magnification of 2 kx and magnification of 20 kx).

Supplement: Supplemental data [file Suppl_FigS2.docx]

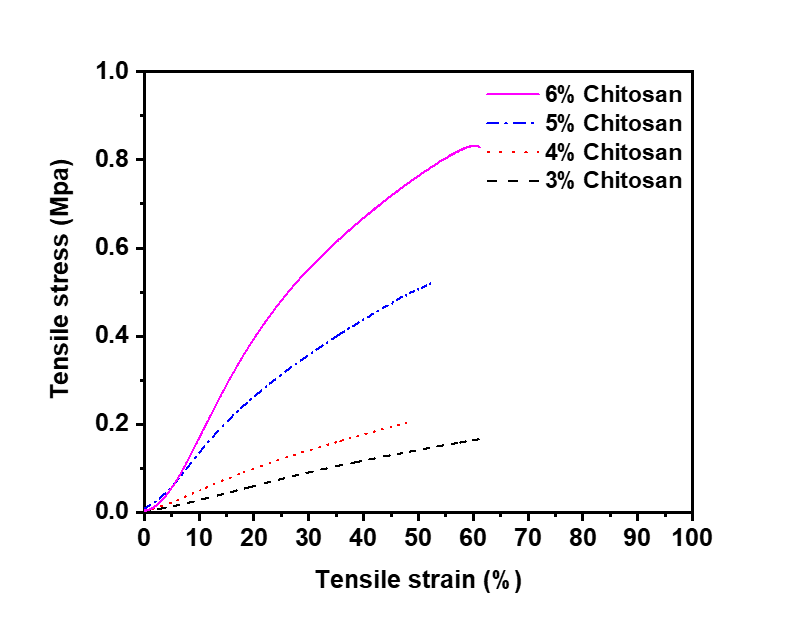


**Figure S3.** Stress-strain curves of chitosan membranes with different concentration.

Supplement: Supplemental data [file Suppl_FigS3.docx]
