# Supplementary material for: Biodegradable Chitosan-Based Membranes for Highly Effective Separation of Emulsified Oil/Water
Source: Environ Eng Sci. 2022 Dec 13;39(12):907–17. doi: 10.1089/ees.2022.0254 (PMC9807252; doi:10.1089/ees.2022.0254)
Supplement: Supplemental data [file Suppl_FigS4.docx]

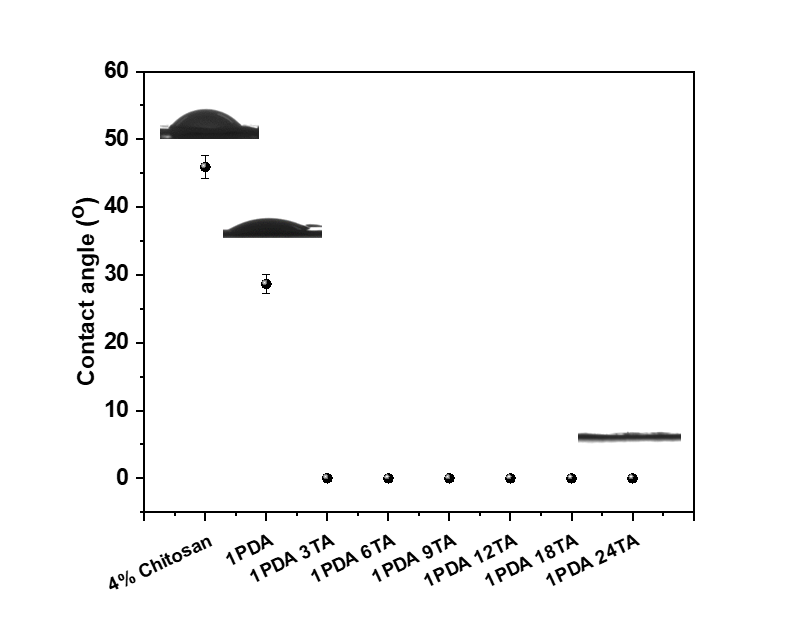


**Figure S4.** Pure water contact angles of 4% chitosan membrane, dopamine-modified membrane, and membranes further modified by TA for different durations.
